# Relação da Função Pulmonar e da Força Inspiratória com Capacidade Aeróbica e com Prognóstico na Insuficiência Cardíaca

**DOI:** 10.36660/abc.20201130

**Published:** 2022-01-11

**Authors:** Sergio Henrique Rodolpho Ramalho, Alexandra Correa Gervazoni Balbuena de Lima, Fabiola Maria Ferreira da Silva, Fausto Stauffer Junqueira de Souza, Lawrence Patrick Cahalin, Graziella França Bernardelli Cipriano, Gerson Cipriano

**Affiliations:** 1 Programa de Pós-graduação em Ciências e Tecnologias em Saúde Universidade de Brasília Brasília DF Brasil Programa de Pós-graduação em Ciências e Tecnologias em Saúde - Universidade de Brasília, Brasília,DF - Brasil; 2 Programa de Pós-graduação em Ciências da Reabilitação Universidade de Brasília Brasília DF Brasil Programa de Pós-graduação em Ciências da Reabilitação - Universidade de Brasília,Brasília, DF - Brasil; 3 Universidade de Miami Miller School of Medicine Miami EUA Universidade de Miami - Miller School of Medicine,Miami - EUA

**Keywords:** Insuficiência Respiratória, Músculos Respiratórios, Função Ventricular, Tolerância ao Exercício, Medição de Risco

## Abstract

**Fundamento:**

A espirometria é subutilizada na insuficiência cardíaca (IC) e não está claro o grau de associação de cada defeito com a capacidade de exercício e com o prognóstico desses pacientes.

**Objetivo:**

Determinar a relação da %CVF prevista (ppCVF) e do VEF_1_/CVF contínuos com: 1) pressão inspiratória máxima (PImáx), fração de ejeção do ventrículo esquerdo (FEVE) e desempenho ao exercício; e 2) prognóstico, para o desfecho composto de morte cardiovascular, transplante cardíaco ou implante de dispositivo de assistência ventricular.

**Métodos:**

Coorte de 111 participantes com IC (estágios AHA C/D) sem pneumopatia; foram submetidos a espirometria, manovacuometria e teste cardiopulmonar máximo. As magnitudes de associação foram verificadas por regressões lineares e de Cox (HR; IC 95%), ajustadas para idade/sexo, e p <0,05 foi considerado significativo.

**Resultados:**

Com idade média 57±12 anos, 60% eram homens, 64% em NYHAIII. A cada aumento de 10% no VEF_1_/CVF [β 7% (IC 95%: 3-10)] e no ppCVF [4% (2-6)], foi associado à reserva ventilatória (VRes); no entanto, apenas o ppCVF associado à PImáx [3,8cmH_2_O (0,3-7,3)], à fração de ejeção do ventrículo esquerdo (FEVE) [2,1% (0,5-3,8)] e ao VO_2_ pico [0,5mL/kg/min (0,1-1,0)], considerando idade/sexo. Em 2,2 anos (média), ocorreram 22 eventos; tanto FEV1/FVC (HR 1,44; IC 95%: 0,97-2,13) quanto ppCVF (HR 1,13; 0,89-1,43) não foram associados ao desfecho. Apenas no subgrupo FEVE ≤50% (n=87, 20 eventos), VEF_1_/CVF (HR 1,50; 1,01-2,23), mas não ppCVF, foi associado a risco.

**Conclusão:**

Na IC crônica, ppCVF reduzido associou-se a menor PImáx, FEVE, VRes e VO_2_ pico, mas não distinguiu pior prognóstico em 2,2 anos de acompanhamento. Entretanto, VEF_1_/CVF associou-se apenas com VRes, e, em participantes com FEVE ≤50%, o VEF_1_/CVF reduzido mostrou pior prognóstico proporcional. Portanto, VEF_1_/CVF e ppFVC contribuem para melhor fenotipagem de pacientes com IC.

## Introdução

A insuficiência cardíaca (IC) e a disfunção pulmonar frequentemente coexistem, emergindo de vários mecanismos: espessamento septal e congestão parenquimatosa; redução da função vascular pulmonar e hipoperfusão microvascular; desregulação e remodelação das vias aéreas; fraqueza muscular inspiratória e periférica; desequilíbrio dos quimio, ergo e metaborreflexos para controle ventilatório; cardiomegalia; e diminuição da condutância brônquica.^1-3^ No entanto, a espirometria é amplamente subutilizada na IC. Mesmo quando IC e doença pulmonar obstrutiva crônica (DPOC) coexistem, 80% dos indivíduos realizam ecocardiografia, mas <50% realizam espirometria.^4-6^

Alterações ventilatórias subclínicas estão presentes nos estágios iniciais da IC, contribuindo para dispneia e intolerância ao exercício.^3^ A obstrução das vias aéreas pode ser encontrada principalmente em pacientes não compensados, e defeitos restritivos são descritos, sobretudo, em indivíduos crônicos e estáveis.^7,8^ O reconhecimento de alterações espirométricas de base fundamenta a interpretação do teste de exercício cardiopulmonar (CPX) para o diagnóstico diferencial de limitação de esforço,^8,9^ e também identifica o risco de mortalidade em IC com fração de ejeção preservada (ICFEP) ou IC com fração de ejeção reduzida (ICFER).^10,11^

No entanto, a associação dos parâmetros espirométricos com a limitação do exercício e prognóstico na IC ainda é controversa,^8^ dado o seu uso em diferentes fenótipos e estados de gravidade da IC, a possível contribuição diferencial de cada defeito obstrutivo e restritivo e as potenciais relações não lineares e pouco exploradas entre as disfunções pulmonares e cardíacas.^12^ Nossa hipótese é que, na IC estável crônica, o comprometimento da capacidade vital forçada (CVF) e da relação do volume expirado forçado em 1 segundo (VEF_1_)/CVF se associam de forma diferente com outros parâmetros funcionais em repouso e no exercício e, consequentemente, com pior prognóstico. Portanto, os objetivos foram (1) definir até que ponto o VEF_1_/CVF e CVF se associam com a fração de ejeção do ventrículo esquerdo (FEVE), força respiratória e respostas ao exercício; e (2) determinar suas associações com a incidência de eventos cardiovasculares maiores (morte cardiovascular, transplante cardíaco e dispositivo de assistência ventricular esquerda (DAVE).

## Métodos

### População do estudo e características clínicas

Esta coorte incluiu 158 pacientes consecutivos com IC encaminhados ao laboratório de fisiologia (Universidade de Brasília, Brasília, Brasil) para CPX de junho de 2015 a julho de 2016, que foram seguidos até julho de 2018, correspondendo a pelo menos 24 meses de acompanhamento. Pacientes diagnosticados com IC, independentemente da etiologia ou FEVE, deveriam estar clinicamente estáveis por pelo menos 3 meses antes da inclusão no estudo (sem descompensação ou hospitalização), sem doença pulmonar diagnosticada (asma, DPOC, enfisema ou em uso de broncodilatadores), sem condições clinicas que impossibilitassem um CPX máximo em cicloergômetro. Os participantes foram submetidos à ecocardiograma (HD 11XE, Phillips, Amsterdam, Holanda) até 1 mês antes da inclusão. Para esta análise, incluímos 111 pacientes com IC, considerando dados espirométricos incompletos em 43 participantes, e 4 não conseguiram manobras com qualidade mínima para interpretação adequada.^13^

No primeiro dia, os indivíduos foram submetidos à avaliação clínica, seguida de avaliação da força respiratória e espirometria após 30 minutos de repouso. O CPX foi realizado no dia seguinte. O ecocardiograma foi realizado de acordo com as recomendações;^14^ a pressão sistólica da artéria pulmonar (PSAP) foi estimada a partir da velocidade de pico do jato de regurgitação tricúspide com Doppler, quando disponível. Hipertensão e diabetes foram definidos com base no autorrelato, uso de medicamentos ou medidas elevadas na consulta (pressão arterial ≥140/90 mmHg e glicemia de jejum ≥126 ou glicose aleatória ≥200mg/dL, respectivamente). Dislipidemia foi definida como LD L≥160mg/dL ou uso de hipolipemiante. O tabagismo foi autorrelatado. O cardiologista assistente informou a etiologia primária da IC e a prescrição farmacológica.

Todos os participantes assinaram um termo de consentimento informado e a aprovação do conselho de revisão institucional foi obtida no Comitê de Ética em Pesquisa da Universidade de Brasília (CAAE 50414115.4.0000.0030).

### Avaliação da função pulmonar e força respiratória

A espirometria foi realizada de acordo com as recomendações-padrão.^13^ VEF_1_ foi obtido a partir do volume de gás exalado no primeiro segundo da expiração. A CVF foi obtida a partir do volume de gás vigorosamente exalado após uma inspiração máxima (Microlab, Carefusion, Yorba Linda, USA). A melhor de cinco tentativas foi utilizada. Os parâmetros de referência derivaram de equações brasileiras.^15^ O VEF_1_/CVF e o percentual do previsto da CVF (ppCVF) foram considerados as principais exposições primárias como variáveis contínuas na análise principal. Em análise de sensibilidade, também analisamos os tercis de cada padrão espirométrico e, ainda, a dicotomização em padrões obstrutivo e não obstrutivo (VEF_1_/CVF≤70 e >70, respectivamente) e padrões restritivos e não restritivos (ppCVF <80% e ≥80%, respectivamente). A força muscular inspiratória (PImáx) e expiratória (PEmáx) máximas foram medidas de acordo com as recomendações,^16^ obtidas com um transdutor digital (MVD300, Globalmed, Porto Alegre, Brasil). Os participantes estavam na posição sentada usando clipe nasal e bocal. A PImáx foi determinada com um esforço inspiratório máximo a partir do volume mais próximo possível ao residual, contra uma via aérea ocluída, com escape aéreo mínimo (2mm). A PEmáx foi determinada com um esforço expiratório máximo a partir do mais próximo possível da capacidade, contra uma via aérea obstruída. Foram realizadas três a cinco manobras reprodutíveis (≤10% da variação entre os valores), sustentadas por pelo menos 1 segundo cada; foram separadas por 1 minuto de descanso e o maior valor foi utilizado para análise.^16^ A PImáx baixa foi considerada quando a PImáx era ≤80cmH_2_O em homens e ≤60cmH_2_O em mulheres.^17^

### Teste de esforço cardiopulmonar

Os pacientes foram submetidos a um CPX máximo, sintoma-limitado,^18^ usando o protocolo de rampa com cicloergômetro (Corival, Lode, Holanda) e um analisador metabólico de gases expirados (Quark CPET, Cosmed, Itália). Calibração de gases e volumes foi realizada antes de cada teste. A ventilação-minuto (VE), o consumo de oxigênio (VO_2_) e a produção de dióxido de carbono (VCO_2_) foram adquiridos respiração por respiração e a média foi calculada em intervalos de 10 segundos. O limiar anaeróbio ventilatório (LAV) foi determinado pelo método V-*slope*. O VO_2_ de pico foi definido como a maior média de 10 segundos durante o platô final, se o paciente o atingiu, ou a maior média de 20 segundos no minuto final do teste sintoma-limitado. O VE/VCO_2_-*slope* foi calculado a partir de uma regressão linear, entre a VE e a VCO_2_ desde o início do teste até o pico do exercício. Devido à fadiga relatada nos testes preliminares de VVM (ventilação voluntária máxima) (não exibidos), subestimando resultados de manobras forçadas subsequentes ou limitando a realização de medida reprodutível, especialmente nos pacientes mais avançados, não conseguimos utilizar a VVM como padrão em toda a coorte, para garantir a comparabilidade. Portanto, a reserva ventilatória foi estimada a partir do VEF_1_ (calculada como 100-[VE/(VEF_1×_40)×100]).^18^ A potência circulatória foi calculada a partir do produto do VO_2_ pico e pressão arterial sistólica máxima e a potência ventilatória calculada pelo quociente entre a pressão arterial sistólica máxima e a Ve/VCO_2_-*slope.*^19^

### Eventos incidentais

O desfecho incidental foi composto de mortalidade cardiovascular, transplante cardíaco de urgência ou implantação de DAVE após a inclusão no estudo. A vigilância dos eventos ocorreu a cada 3 meses, por meio telefônico, revisando prontuários hospitalares ou confirmação de registros de atestados de óbito.

### Abordagem estatística

As características gerais foram descritas usando média e desvio padrão para variáveis contínuas e números absolutos e porcentagens para variáveis categóricas. Kolmogorov-Smirnov foi utilizado e todas as variáveis mostraram distribuição normal. Para a análise transversal, foi utilizada regressão linear para avaliar associações entre VEF_1_/CVF e ppCVF, como variáveis de exposição contínuas, com estrutura cardíaca, força respiratória e parâmetros do CPX como variáveis dependentes, em modelos não ajustados e ajustados por idade e sexo, exibidos como coeficiente-ß e intervalo de confiança 95% (95%IC), para cada 10 pontos percentuais de aumento no parâmetro espirométrico. Considerando a distribuição normal e a independência das observações dentro de cada modelo (correlação de Pearson <0,35 entre cada exposição e desfecho), as premissas foram verificadas para a regressão linear. Para abordar potenciais associações não lineares, também testamos modelos polinomiais (*splines*) cúbicos restritos usando 3 a 7 nós, não ajustados e ajustados para idade e sexo.

Para análise de sensibilidade, as variáveis de exposição também foram categorizadas em: a) tercis sexo-específicos para VEF_1_/CVF e ppCVF, sendo o primeiro tercil representando a pior função pulmonar, e o terceiro tercil, a melhor função pulmonar; e b) grupos dicotômicos, comparando padrões obstrutivos (VEF_1_/CVF ≤70) com não obstrutivos (VEF_1_/CVF >70) e padrões restritivos (ppCVF <80%) com não restritivos (ppCVF ≥80%). Regressões lineares e logísticas e teste de Qui-quadrado para tendência foram usados para avaliar as associações. Para verificar possíveis assimetrias entre participantes incluídos e excluídos, esses grupos foram comparados usando o Qui-quadrado para variáveis categóricas e teste *t* para amostras independentes para contínuas.

Para a análise prospectiva, regressão de Cox foi utilizada para determinar a magnitude da associação entre a redução de 10 pontos percentuais na função pulmonar com o a incidência do desfecho composto, mostrado como *hazard ratio* (HR) e IC 95%. Associações não lineares foram investigadas usando regressão cúbica restrita (*spline*) com o número de nós selecionados para minimizar o modelo AIC (3 a 7 nós testados). A presunção de riscos proporcionais foi testada para todos os modelos usando resíduos de Schoenfeld, e nenhuma violação foi detectada.

Para a análise prospectiva, a regressão de Cox foi usada para determinar a magnitude da associação da diminuição de 10 pontos percentuais na variável da espirometria com o desfecho composto incidental, mostrado como razão de risco (HR) e IC 95%. As associações não lineares foram investigadas usando regressão spline cúbica restrita com o número de nós selecionados para minimizar o modelo AIC (3 a 7 nós testados). A suposição de riscos proporcionais foi testada para todos os modelos usando resíduos de Schoenfeld, e nenhuma violação foi detectada. Como abordagem de sensibilidade, regressões de Cox foram realizadas restringindo 4 subgrupos: FEVE ≤50%; FEVE >50%; PImáx baixa; e PImáx normal.

Um valor de p bicaudal <0,05 foi considerado significativo para todas as análises. Foi utilizado o programa STATA versão 14.2 (Stata Corp LP, College Station, Texas, EUA).

## Resultados

Entre os 111 participantes com IC, a etiologia isquêmica foi predominante, estágios American Heart Association C ou D, tratados de acordo com as diretrizes, dentre os quais 24 apresentaram FEVE >50% (Tabela 1). Aproximadamente metade dos indivíduos apresentava um padrão restritivo (ppCVF <80%); um quarto, um padrão obstrutivo (VEF_1_/CVF≤70); e 14 indivíduos (13%), disfunções combinadas; enquanto, em 40 deles (36%), a espirometria era normal. Dos 26 (23%) pacientes com índice de massa corporal (IMC) maior que 30kg/m^2^, 15 (65%) apresentaram um ppCVF <80%. Entre 57 pacientes com ppCVF <80% (51%), 15 tinham IMC >30kg/m^2^. Baixa PImáx foi um achado frequente. O VO_2_ de pico médio foi baixo, mesmo garantindo critérios de esforço máximo. A fadiga muscular geral ou a de membros inferiores foram sintomas limitantes, não houve sibilância ou cianose. Cinco pacientes apresentaram reserva ventilatória inferior a 20%, dentre eles 4 com 10% a 15%; com distúrbios basais restritivos (3) ou combinados (2) e FEVE <34%. Entre estes, o intervalo RQ foi 1,09 e 1,22. Aqueles não incluídos por falta de dados ou por espirometria de má qualidade apresentaram características semelhantes aos incluídos, exceto pela média de idade mais jovem (51,6±14,2 anos) (Tabela Suplementar S1).

**Tabela 1:** – Características basais da população com insuficiência cardíaca (n=111). Valores são mostrados como média ± DP ou n (%)

Participantes, n	111
**Dados demográficos e clínicos**	
Idade, anos	57,4 ± 11,8
Masculino, n(%)	67 (60%)
**Etiologia, n(%)**	
Chagas	32 (29%)
Isquêmica	43 (39%)
Idiopática	23 (21%)
Outra	13 (12%)
IMC, kg/m^2^	26,6 ± 4,8
IMC >30 kg/m^2^; n(%)	26 (23%)
**História médica**	
Hipertensão, n(%)	63 (57%)
Diabetes, n(%)	20 (18%)
Fumantes ativos, n(%)	29 (26%)
Dislipidemia, n(%)	44 (40%)
NYHA, n(%)	
I	15 (13%)
II	25 (22%)
III	71 (64%)
**Medicações e dispositivos**	
Betabloqueadores, n(%)	100 (90%)
IECA/BRA, n(%)	94 (84%)
Espironolactona, n(%)	73 (66%)
Digoxina, n(%)	22 (20%)
Estatina, n(%)	70 (63%)
Furosemida, n(%)	67 (60%)
Marca-passo/CDI, n(%)	25 (22%)
**Função pulmonar**	
VEF_1_, L	2.3 ± 0.7
CVF, L	3.0 ± 0.9
Porcentagem prevista da CVF, %	80 ± 17
Porcentagem prevista da CVF <80%	57 (51%)
VEF_1_/CVF	75 ± 9
VEF_1_/CVF ≤70	28 (25%)
PEmáx, cmH_2_O	84,7 ± 40,1
PImáx, cmH_2_O	75,4 ± 35,4
PImáx reduzida, n(%)	51 (49%)
**Ecocardiograma**	
FEVE, %	38,4 ± 15,0
FEVE >50%, n(%)	24 (23%)
Volume do AE indexado, mL/m^2^	44,7 ± 16,7
PSAP estimada, mmHg	38,9 ± 12,0
**Teste cardiopulmonar**	
Potência de pico, W	80,3 ± 30,6
Frequência cardíaca de pico, bpm	118 ± 26
Pressão sistólica de pico, mmHg	151 ± 25
VO_2_ absoluto de pico, mL/min	966 ±401
VO_2_ relativo de pico, mL/kg/min	13,4 ± 4,6
RER	1,23 ± 0,18
VO_2_ absoluto no LAV, mL/min	618 ± 281
VO_2_ relativo no LAV, mL/kg/min	8,6 ± 3,5
Pulso de O_2_, mL/batimento	8.4 ± 3.1
OUES	1145 ± 465
VE max, (L/min)	45.0 ± 16.6
Reserva ventilatória, %	48 ± 19
VE/VCO_2_*slope*	37.3 ± 8.1
Poder circulatório, mmHg.mL/kg/min	2165 ± 1024
Poder ventilatório, mmHg	4.3 ± 1.4

*IMC: índice de massa corporal; VEF_1_: volume expirado forçado em 1 segundo; CVF: capacidade vital forçada; NYHA: classe funcional da New York Heart Association; IECA: inibidores da enzima de conversão da angiotensina; BRA: bloqueadores do receptor da angiotensina II; CDI: cardioversor desfibrilador implantável; PEmáx: pressão expiratória máxima; PImáx: pressão inspiratória máxima; FEVE: fração de ejeção do ventrículo esquerdo do ventrículo esquerdo; AE: átrio esquerdo; PSAP: pressão sistólica da artéria pulmonar; VO_2_: consumo de oxigênio; OUES: curva da eficiência de captação de oxigênio; VE: ventilação minuto; VE/VCO_2_ slope: VE/produção de dióxido de carbono; RER: razão de troca respiratória; LAV: limiar anaeróbio ventilatório.*

### Relação do VEF1/CVF com variáveis funcionais e com prognóstico

**Figura 1:**
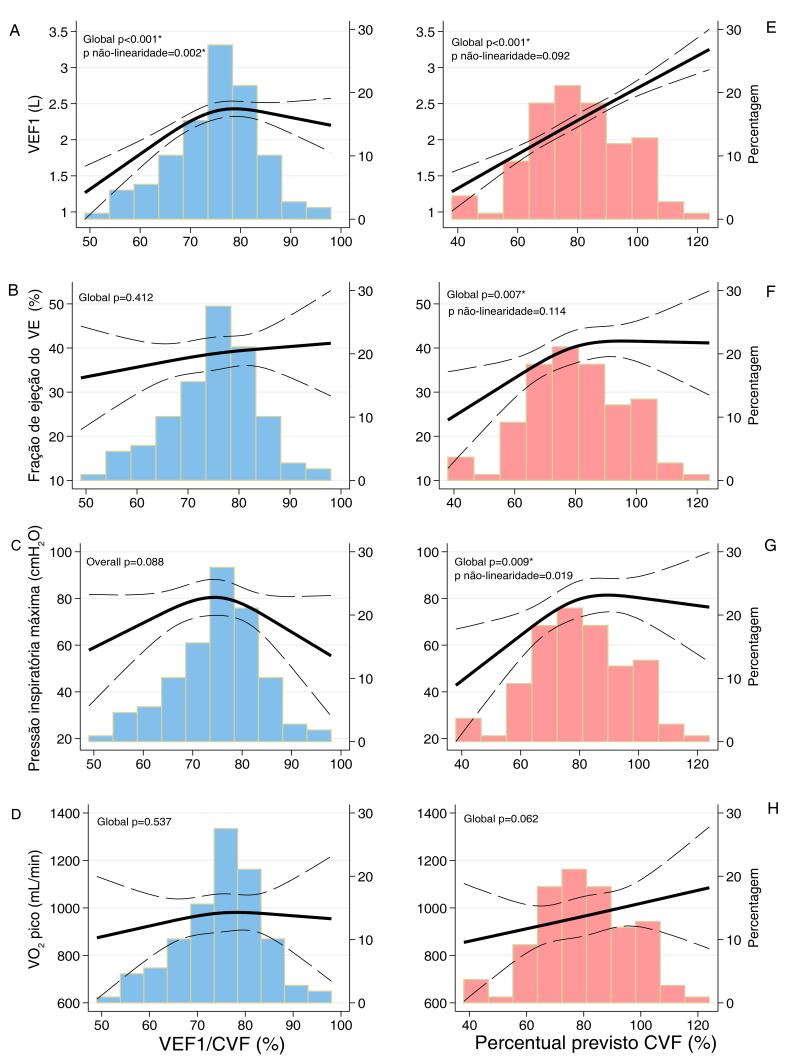
– Associação contínua da VEF_1_/CVF (azul) e do percentual previsto da CVF (vermelho-claro) com VEF_1_, FEVE, PImáx e VO_2_ de usando splines cúbicos restritos. Modelos foram construídos usando splines cúbicos restritos com 3 nós. *p <0,05 em modelos adicionalmente ajustados para idade e sexo.

Em modelos contínuos, o VEF_1_/CVF foi proporcionalmente associado ao VEF_1_ e à reserva ventilatória pelo CPX, de modo que, a cada aumento de 10 pontos percentuais no VEF_1_/CVF, houve aumento de 200mL (IC 95% 100-310mL, p <0,001) em VEF_1_ e de 7 pontos percentuais (IC 95% 3-10%; p <0,001) de aumento na reserva ventilatória, após ajuste para idade e sexo (Tabela 2). Embora a PImáx baixa fosse um achado comum em indivíduos com padrão obstrutivo (n=15, 54%), a frequência foi semelhante quando comparada ao padrão não obstrutivo (n=36, 47%; p=0,54), e a PImáx não foi associada ao VEF_1_/CVF contínuo (p=0,90). Além disso, foi observada uma associação não linear entre VEF_1_/CVF e VEF_1_, de forma que essa relação é mais robusta se VEF_1_/CVF for inferior a 75% (Figura 1A). Nenhuma outra métrica de estrutura ou função cardiopulmonar foi associada com VEF_1_/CVF. Esses achados foram consistentes também entre os tercis VEF_1_/CVF (Tabela Suplementar S2).

**Tabela 2:** – Relação contínua entre os parâmetros espirométricos (por aumento de 10 pontos percentuais em cada VEF1/CVF e ppCVF) e a função cardiopulmonar em participantes com insuficiência cardíaca

Função cardíaca e pulmonar		VEF_1_/CVF		% prevista da CVF	

		Coeficiente (95%IC)	p	Coeficiente (95%IC)	p
VEF_1_, *L*	Modelo 1	0,24 (0,10; 0,38)	0,001	0,25 (0,18; 0,31)	<0,001
	Modelo 2	0,20 (0,10; 0,31)	<0,001	0,23 (0,19; 0,27)	<0,001
PEmáx *cmH*_*2*_*O*	Modelo 1	2,7 (-6,6; 11,9)	0,57	-0,9 (-5,8; 4,1)	0,73
	Modelo 2	0,6 (-7,5;8,8	0,88	-1,5 (-5,8; 2,9)	0,50
PImáx, *cmH*_*2*_*O*	Modelo 1	0,9 (-0,6; 8,2)	0,81	4,1 (0,2; 8,1)	0,04
	Modelo 2	0,4 (-6,1; 6,9)	0,90	3,8 (0,3; 7,3)	0,031
FEVE, *%*	Modelo 1	1,9 (-1,0; 4,9)	0,20	2,2 (0,6; 3,9)	0,007
	Modelo 2	1,6 (-1,4; 4,7)	0,28	2,1 (0,5; 3,8)	0,013
Potência pico, *W*	Modelo 1	1,5 (-4,6; 7,6)	0,63	4,2 (0,9; 7,5)	0,012
	Modelo 2	0,3 (-4,3; 4,9)	0,89	3,5 (1,1; 6,0)	0,005
VO_2_ absoluto de pico, *mL/min*	Modelo 1	30 (-50; 111)	0,45	35 (-9; 78)	0,11
	Modelo 2	18 (-49; 86)	0,59	27 (-9; 63)	0,14
VO_2_ relativo de pico, *mL/kg/min*	Modelo 1	-0,3 (-1,3; 0,6)	0,47	0,6 (0,1; 1,1)	0,02
	Modelo 2	-0,4 (-1,3; 0,4)	0,30	0,5 (0,1; 1,0)	0.028
Razão de troca ventilatória	Modelo 1	-0,01 (-0,05; 0,02)	0,48	0,02 (-0,003; 0,04)	0,09
	Modelo 2	-0,01 (-0,05; 0,02)	0,40	0,01 (-0,003; 0,03)	0,10
VO_2_ absoluto no LAV, *mL/min*	Modelo 1	-8 (-66; 49)	0,77	12 (-20; 44)	0,45
	Modelo 2	-12 (-67; 44)	0,68	13 (-18; 44)	0,42
VO_2_ relativo no LAV, *mL/kg/min*	Modelo 1	-0,7 (-1,4; -0,004)	0,05	0,2 (-0,2; 0,6)	0,31
	Modelo 2	-0,7 (-1,4; 0,007)	0,05	0,2 (-0,2; 0,6)	0,25
Pulso de O_2_, *mL/batimento*	Modelo 1	0,4 (-0,2; 1,0)	0,21	0,1 (-0,2; 0,5)	0,45
	Modelo 2	0,4 (-0,2; 0,9)	0,18	0,1 (0,2; 0,4)	0,47
OUES	Modelo 1	62 (-31; 155)	0,19	19 (-32; 70)	0,47
	Modelo 2	48 (-34; 130)	0,25	9 (-36; 54)	0,70
VE máx, *L/min*	Modelo 1	-0,4 (-3,7; 2,9)	0,82	1,6 (-0,2; 3,4)	0,08
	Modelo 2	-0,6 (-3,3; 2,1)	0,68	1,5 (0,02; 2,9)	0,05
Reserva ventilatória, *%*	Modelo 1	7,4 (3,9; 10,9)	<0,001	4,6 (2,8; 6,5)	<0,001
	Modelo 2	6,8 (3,3; 10,3)	<0,001	4,3 (2,5; 6,2)	<0,001
VE/VCO_2_*slope*	Modelo 1	-0,2 (-2,0; 1,7)	0,87	-0,6 (-1,6; 0,3)	0,19
	Modelo 2	0,2 (-1,6; 2,0)	0,85	-0,5 (-1,5; 0,5)	0,35
Pot. circulatória, *mmHg.mL/kg/min*	Modelo 1	-20 (-202; 163)	0,83	85 (-14; 183)	0,09
	Modelo 2	-43 ( -212; 125)	0,61	72 (-19; 163)	0,12
Pot. ventilatória, *mmHg*	Modelo 1	0,08 (-0,20; 0,35)	0.59	0.12 (-0,03; 0,27)	0,11
	Modelo 2	0,04 (-0,22; 0,31)	0.76	0.10 (-0,04; 0,24)	0,17

*VEF_1_: volume expirado forçado em 1 segundo; CVF: capacidade vital forçada; PEmáx: pressão expiratória máxima; PImáx: pressão inspiratória máxima; FEVE: fração de ejeção do ventrículo esquerdo do VE; VO_2_: consumo de oxigênio; LAV: limiar anaeróbio ventilatório; OUES: curva da eficiência de captação de oxigênio; VE: ventilação minuto; VCO_2_: produção de dióxido de carbono; Pot.: potência. Modelo 1: não ajustado; Modelo 2: idade e sexo. Nota: valores de p se referem à respectiva análise de regressão linear.*

**Figura 2:**
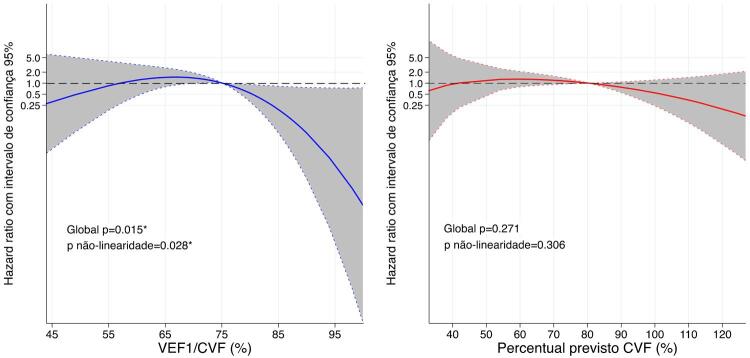
– Associações contínuas de VEF_1_/CVF (azul) e percentual previsto da CVF (vermelho) basais com o desfecho composto (morte cardiovascular, transplante cardíaco e implante de dispositivo de assistência ventricular), em tempo médio de acompanhamento de 2,2±0,7 anos (22 eventos). Modelos foram construídos para a variável primária de exposição (VEF_1_/CVF e percentual previsto da CVF) usando splines cúbicos restritos com 3 nós. Linear corresponde à análise de regressão de Cox. *p <0,05 em modelos adicionalmente ajustados para idade e sexo.

No seguimento médio de 2,2±0,7 anos, 15 indivíduos tiveram morte cardiovascular, 3 foram transplantados e 4 tiveram implante de DAVE. Houve tendência de menor VEF_1_/CVF aumentar o risco para o desfecho composto, porém não foi linearmente significativo quando considerados a idade e o sexo (Tabela 3). Por outro lado, houve associação não linear entre VEF_1_/CVF e o desfecho composto, de forma que o risco diminui quando VEF_1_/CVF >75 (Figura 2).

**Tabela 3:** – Associação das variáveis espirométricas basais com a incidência do desfecho composto (mortalidade cardiovascular, transplante cardíaco e implante de dispositivo de assistência ventricular esquerda; 22 eventos) entre participantes com insuficiência cardíaca (n=111), acompanhados por 2,2±0,7 anos

	n	Eventos	Não ajustado HR (95%IC)*	p	Ajuste por sexo e idade HR (95%IC)*	p
**VEF**_**1**_**/CVF**						
Obstrutivo	28	9	2,45 (1,05-5,77)	p=0,039	2,28 (0,95-5,44)	p=0,064
Não obstrutivo	83	13				
Contínuo	111	22	1,48 (1,00-2,18)	p=0,050	1,44 (0,97-2,13)	p=0,069
**ppCVF**						
Restritivo	57	14	1,83 (0,77-4,37)	p=0,172	1,86 (0,78-4,44)	p=0,163
Não restritivo	54	8				
Contínuo	111	22	1,16 (0,92-1,46)	p=0,207	1,13 (0,89-1,43)	p=0,306

*por 10 unidades de redução. Nota: valores de p se referem à respectiva análise de regressão de Cox.

Duas análises de sensibilidade foram realizadas. Primeiramente, excluindo aqueles com FEVE >50% (n=24), dentre os 87 sujeitos restantes, 20 eventos ocorreram. Nesse cenário, cada redução de 10 pontos percentuais no VEF_1_/CVF foi associada a um aumento de 50% na probabilidade de o desfecho composto incidir por ano de observação, levando em consideração a idade e o sexo (p=0,04) (Figura Suplementar S1). Entre aqueles com FEVE >50%, apenas dois eventos ocorreram. Em segundo lugar, entre indivíduos com baixa PImáx (n=51, 13 eventos), VEF_1_/CVF reduzido foi associado a maior risco para o desfecho primário (HR 1,72; 1,14-2,61; p=0,009), enquanto no subgrupo com PImáx normal (n=57, seis eventos), não foi associado ao desfecho (HR 0,98; 0,36-2,69) (Figura Suplementar S1)

### Relação da porcentagem da CVF prevista com variáveis funcionais e com prognóstico

Considerando a idade e o sexo, cada aumento de 10 pontos percentuais no ppCVF ajustado foi proporcionalmente associado a um aumento linear no VEF_1_, de 230mL (IC 95% 190-270mL, p <0,001) (Tabela 2). A PImáx também aumentou 3,8cmH_2_O (IC 95% 0,3-7,3, p = 0,03), mas a análise não linear mostrou que essa associação foi mais robusta para ppCVF <80% (Tabela 2 e Figura 1G). A PImáx baixa foi mais frequente em indivíduos com IC com padrão restritivo (n=34, 65%) quando comparados àqueles sem padrão restritivo (n=17, 32%; p <0,001). A FEVE aumentou aproximadamente 2 pontos percentuais para cada aumento de 10 pontos percentuais no ppCVF, que também foi mais proeminente quando ppCVF <80% (Figura 1F). Em relação ao CPX, quanto maior o ppCVF, maior a potência de pico, o VO_2_ de pico relativo e a reserva ventilatória nos modelos ajustados. Nenhuma outra métrica de estrutura ou função cardiopulmonar foi associada ao ppCVF, como variável contínua (Tabela 2) ou categorizada em tercis (Tabela Suplementar S3).

O ppFVC mais baixo não foi capaz de distinguir indivíduos com IC sob maior risco para o desfecho composto na análise primária (Tabela 3 e Figura 2) ou de sensibilidade (Figura Suplementar S2).

## Discussão

**Figura 3:**
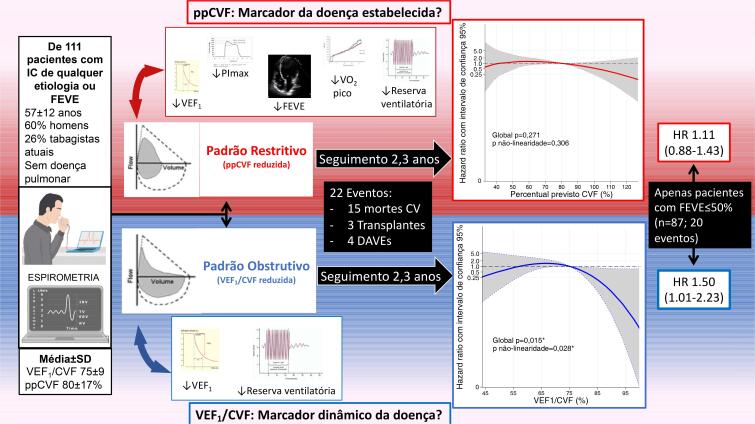
– Resumo visual dos achados principais: VEF_1_/CVF e ppCVF caracterizam de forma diferencial pacientes com insuficiência cardíaca. IC: insuficiência cardíaca; FEVE: fração de ejeção do ventrículo esquerdo; VEF_1_: volume expiratório forçado em 1 segundo; ppCVF: percentual previsto da capacidade vital forçada; PImáx: pressão inspiratória máxima; CV: cardiovascular; DAVE: dispositivo de assistência ventricular esquerda; HR: hazard ratio. Apesar de a ppCVF estar associada a outras variáveis funcionais além da reserva ventilatória, apenas o VEF_1_/CVF foi associado a prognóstico de curto prazo, particularmente para aqueles com fração de ejeção reduzida, sugerindo que cada marcador agrega diferentes informações em relação aos fenótipos da insuficiência cardíaca.

Em uma coorte de mundo real de 111 indivíduos com IC crônica de classes C ou D, dentro de uma ampla faixa de fração de ejeção, investigamos como o espectro de distúrbios espirométricos normal a grave se relacionava com as métricas funcionais de repouso e de exercício, e com eventos cardiovasculares incidentais maiores. Em todas as faixas de obstrução das vias aéreas e de comprometimento da capacidade vital, levando em consideração a idade e o sexo, tanto o VEF_1_/CVF baixo quanto o ppCVF foram associados à redução da reserva ventilatória ao exercício, mas apenas o ppCVF baixo foi associado à menor fração de ejeção, fraqueza inspiratória e menor capacidade de exercício, que foi mais proeminente quando o ppCVF foi inferior a 80%. Embora tais disfunções pulmonares fossem comuns, o risco para o desfecho composto de morte cardiovascular, transplante cardíaco ou implante de DAVE teve associação não linear apenas com VEF_1_/CVF, mas não com ppCVF, sugerindo um melhor prognóstico com padrão não obstrutivo (VEF_1_/CVF >75%). Além disso, entre os subgrupos de baixa FEVE e baixa PImáx, apenas o VEF_1_/CVF reduzido distinguiu um risco maior. Portanto, VEF_1_/CVF e ppCVF fenotipam aspectos clínicos de pacientes com IC de maneira diferente (Figura 3).

### Relação de função pulmonar de repouso com desempenho ao exercício

Os efeitos diretos da IC nas vias aéreas, como congestão vascular, edema parenquimatoso e alveolar e fibrose intersticial, estão relacionados à constrição aguda/subaguda do diâmetro das vias aéreas e à redução subaguda/crônica do volume pulmonar.^1-3^ Como resultado, o VEF_1_ e a CVF diminuem de forma independente ou paralela, sugerindo que mesmo a disfunção subclínica pode contribuir de forma diferente para a intolerância ao exercício. Consequentemente, o VEF_1_ se associa de forma mais robusta à capacidade aeróbia (VO_2_ de pico) do que a FEVE.^8,20^ Portanto, espera-se que a ventilação voluntária máxima (derivada do VEF_1_) seja prejudicada na IC, proporcionalmente à gravidade da disfunção pulmonar subjacente. No entanto, a intolerância ao exercício em indivíduos com IC é multifatorial, e a reserva ventilatória reduzida no pico expressa apenas parcialmente a contribuição pulmonar.^7^ De fato, observamos que a reserva ventilatória aumentou 7 e 4 pontos percentuais para cada aumento de 10 pontos percentuais no VEF_1_/CVF e na ppFVC, respectivamente. No entanto, a reserva ventilatória foi tão baixa quanto 38% e 39%, em média, nos tercis inferiores de VEF_1_/CVF e ppCVF, respectivamente; valores maiores que os 20% esperados para assumir inequivocamente uma restrição ventilatória no pico do exercício, sugerindo a limitação cardiocirculatória como origem primária – mas não única – da limitação de esforço em nossos pacientes.

Parâmetros como OUES e VE/VCO_2_*slope* são menos dependentes do pico de esforço e são mais sensíveis para distinguir restrições ventilatórias de cardiocirculatórias.^20,21^ Os valores médios reduzidos do OUES, elevados de VE/VCO_2_*slope* e reduzidos do pulso de O_2_ em nosso estudo, na verdade, sugerem uma limitação cardiocirculatória predominante, mas a falta de associação entre VEF_1_/CVF e ppCVF em repouso com outras variáveis ventilatórias de exercício, incluindo eficiência ventilatória, foi contrária à nossa hipótese *a priori*. Portanto, a capacidade da espirometria de repouso para determinar precisamente a contribuição ventilatória para limitação ao exercício, além da reserva ventilatória, pode ser limitada, sobrepujada pelo componente cardiocirculatório nos estágios de IC mais avançados, como em nossa coorte, composta de pacientes em estágios C/D, predominantemente em NYHA III (64%) e VO_2_ pico médio de 13mL/kg/min.^22^

Em relação às outras variáveis funcionais, apenas o ppCVF, mas não o VEF_1_/CVF, foi adicionalmente associado à PImáx em repouso, mas não à PEmáx, à FEVE e ao pico de potência e VO_2_ pico relativo, levando em consideração a idade e o sexo.

Possivelmente, dadas as características graves, mas estáveis dos indivíduos com IC neste estudo, as correlações discrepantes para cada exposição poderiam resultar da influência primária da IC na redução da capacidade pulmonar total, diminuindo desproporcionalmente a CVF em relação ao VEF_1_, atenuando, portanto, o efeito do VEF_1_/CVF para prever as respostas do exercício.^1^ Assim, um padrão restritivo é comum na síndrome de IC,^1,7,8^ particularmente em ICFER.^23^ Além disso, a relação direta do ppCVF com a FEVE apoia a hipótese de que um coração potencial aumentado e disfuncional está relacionado ao volume pulmonar reduzido devido aos efeitos de ocupação de espaço e de congestão vascular e parenquimatosa mencionados anteriormente. Tais alterações, agravadas pela fraqueza inspiratória, podem comprometer a mecânica respiratória em resposta às demandas crescentes, reduzindo ainda mais a complacência pulmonar, e todas podem contribuir para a limitação do exercício, representada pelo baixo VO_2_ de pico.^1,7,8^

Curiosamente, apenas o ppCVF foi associado a PImáx. Na IC, a redução da capacidade vital está associada à baixa PImáx e à disfunção diafragmática,^24,25^ que desempenha papel significativo na limitação do exercício na ICFER^26,27^ e na ICFEP^28^ e demonstra relevância prognóstica independente.^29^ Consistentemente com nossos achados, a disfunção e as anormalidades estruturais da musculatura esquelética generalizada, diafragmática em particular, contribuem amplamente para a intolerância ao exercício em ambas, ICFER e ICFEF.^30^ Automaticidade e constante sobrecarga de trabalho, mesmo em estado de repouso, caracterizam unicamente a predisposição do diafragma à disfunção precoce na síndrome de IC, que é mais proeminente que a de outros músculos expiratórios e/ou periféricos.^31^

### Função pulmonar e prognóstico cardiovascular

O declínio da função pulmonar, mesmo subclínico, mostrou-se associado à incidência de insuficiência cardíaca em populações não selecionadas.^32,33^ Além disso, em indivíduos com ICFER estável, a espirometria previu significativamente mortalidade por qualquer causa.^34^ Olson et al.^34^ estudaram 134 indivíduos com ICFER com VO_2_ pico de 19mL/kg/min e 66% eram NYHA classes I e II, e mostraram que quanto menor VEF_1_ ou CVF, menores as taxas de sobrevida. Em contraste, na ICFER avançada pré-transplante, Georgiopoulou et al. demonstraram que a espirometria não forneceu informações prognósticas.^35^ Em nossa coorte, posicionada entre os estudos anteriores quanto à gravidade dos participantes, o VEF_1_/CVF contínuo e o ppCVF não foram capazes de distinguir indivíduos com IC com risco aumentado para eventos cardiovasculares maiores em modelos lineares.

Provavelmente, dados os estágios crônicos da IC e a limitação cardiocirculatória predominante do CPX conforme demonstrado, a contribuição do comprometimento ventilatório forneceu poucas informações prognósticas adicionais considerando todo o espectro de FEVE na IC. Tentando abordar a potencial relação dinâmica desta complexa interação biológica, investigamos associações não lineares para possíveis intervalos ou limites sob risco diferencial, em todo o espectro de ambas as exposições. Encontramos associação não linear entre VEF_1_/CVF e o desfecho composto, em que indivíduos com IC com VEF_1_/CVF maior que 75% diminuíram a probabilidade de apresentar eventos cardiovasculares maiores em uma média de 2,2 anos de acompanhamento. Podemos supor que: 1) a DPOC, a principal causa de obstrução das vias aéreas que frequentemente coexiste com a IC, poderia não ter sido detectada, aumentando a carga de risco;^4^ ou 2) dos efeitos primários da IC no sistema respiratório, as alterações do VEF_1_ podem ser mais sensíveis que a CVF em períodos mais curtos, influenciadas por alterações dinâmicas em brônquios de calibres pequenos e médios.^23^ A capacidade vital reduzida, como marca registrada da IC avançada,^9^ foi menos sensível para distinguir o risco de eventos incidentais ao longo deste tempo de acompanhamento.

Contribuições potenciais da função pulmonar para a incidência de eventos cardiovasculares também parecem diferir entre os fenótipos ICFEP e ICFER. Restringindo a análise a um subconjunto de indivíduos com LVEF ≤50%, uma diminuição no VEF_1_/CVF, mas não na ppCVF, identificou maior risco para o desfecho composto, enquanto nenhuma conclusão pôde ser feita para aqueles com FEVE >50% com apenas dois eventos. Da mesma forma, no subconjunto de fraqueza inspiratória, a redução do VEF_1_/CVF distinguiu maior risco de eventos cardiovasculares maiores, o que não foi observado entre aqueles sem fraqueza ou em todo o espectro ppCVF. Poderíamos especular que a fisiopatologia do padrão obstrutivo impactou negativamente principalmente aqueles com FEVE reduzida e com fraqueza inspiratória.

### Limitações

Várias limitações devem ser observadas. Como um estudo observacional, a causalidade não pôde ser abordada e podem existir fatores de confusão residuais e não medidos para as observações descritas. Apenas um subconjunto de pacientes foi incluído, com espirometria completa, necessária para este estudo e, dada a idade mais jovem daqueles excluídos, viés de seleção involuntário poderia estar presente. As medidas espirométricas foram realizadas sem broncodilatadores, de forma que a obstrução reversível permaneceu indetectável; além disso, os padrões restritivos foram baseados apenas na CVF, porque medidas mais precisas e diretas de volumes e capacidades não estavam disponíveis, o que poderia ter limitado a capacidade de detectar a verdadeira restrição de volume pulmonar, porém poderia aumentar a validade externa dos achados. Além disso, um mecanismo importante de limitação do exercício pode ser devido ao aprisionamento de ar, que não pode ser detectado pela espirometria.

Infelizmente, a VVM medida não foi viável para todos os participantes, o que pode ter influenciado métricas de reserva ventilatória, provavelmente subestimadas, embora apenas 5 participantes tiveram <20% de reserva ventilatória. No entanto, trocamos resultados potencialmente não confiáveis, por valores uniformes e comparáveis em toda a coorte usando a medida de MVV estimada. Apesar de uma correlação razoável entre FEV_1_ e MVV (r^2^ = 0,82),^36^ reconhecemos que o VVM deve ser realizado antes do CPX em nível individual, sempre que possível.

Também reconhecemos que a baixa saturação de oxigênio durante o exercício pode representar um desacoplamento de ventilação-perfusão ou restrição ventilatória, o que não é exclusivamente, mas mais frequentemente associada ao comprometimento do sistema respiratório, particularmente DPOC avançada, doenças pulmonares intersticiais e hipertensão pulmonar,^18^ condições excluídas no início do estudo. No entanto, um oxímetro compatível com nosso sistema de CPX estava indisponível durante a aquisição de dados, e o transdutor digital existente produziu valores não confiáveis. Portanto, recorremos ao exame físico normal (sem sibilância ou cianose no pico de esforço) para supor que a hipóxia era improvável.

Por fim, o tempo de acompanhamento relativamente curto e, consequentemente, a taxa de eventos podem ter limitado a capacidade de detectar associações prognósticas com ppCVF, mas não com VEF_1_/CVF, devido ao comportamento mais crônico do primeiro.

### Implicações para a prática clínica

A espirometria e a manovacuometria são ferramentas de função pulmonar amplamente disponíveis, embora subutilizadas na IC.^4,26^ A interpretação de defeitos ventilatórios pode ser desafiadora nesses indivíduos, particularmente naqueles com ICFEP, cuja variação fenotípica pode sobrepor sintomas cardíacos e pulmonares, e menos dados são disponíveis.^9^ No entanto, podem fornecer valiosas informações sobre o impacto da IC no sistema respiratório, diferenciando-se da doença pulmonar não diagnosticada (e subtratada), interpretando melhor a CPX, identificando potenciais alvos terapêuticos (reabilitação, treinamento ventilatório) e definindo fatores prognósticos, enfatizando que a espirometria é uma ferramenta disponível e viável, que deve ser realizada previamente ao CPX e para apoiar a estratificação de risco na IC. Alterações subclínicas e em estágios iniciais da função pulmonar podem predizer eventos cardiovasculares futuros. Somando-se a esse conhecimento, nosso estudo sugere que, também na IC mais crônica e estável, a presença e o tipo de disfunção pulmonar auxiliam na melhor interpretação das respostas ao exercício e na identificação de sujeitos sob maior risco.

## Conclusão

Em uma coorte do mundo real com indivíduos com IC crônica, com ampla faixa de fração de ejeção, VEF_1_/CVF e ppCVF foram diretamente associados à reserva ventilatória no exercício, levando em consideração a idade e o sexo. No entanto, apenas ppCVF reduzida foi adicionalmente associada a baixa fração de ejeção, fraqueza inspiratória e baixa capacidade de exercício. Em seguimento médio de 2,2 anos, apenas o VEF_1_/CVF, mas não a ppCVF, distinguiu indivíduos com IC sob maior risco de eventos cardiovasculares maiores, sendo mais proeminentes entre aqueles com fração de ejeção reduzida e baixa pressão inspiratória. Portanto, VEF_1_/CVF e ppCVF adicionam informações distintas sobre os fenótipos de IC.
