# Supplementary material for: Relação da Função Pulmonar e da Força Inspiratória com Capacidade Aeróbica e com Prognóstico na Insuficiência Cardíaca
Source: Arq Bras Cardiol. 2022 Jan 11;118(4):680–91. [Article in Portuguese] doi: 10.36660/abc.20201130 (PMC9006999; doi:10.36660/abc.20201130)
Supplement: Translation

# Translation: Relationship of Lung Function and Inspiratory Strength with Exercise Capacity and Prognosis in Heart Failure

**Authors:** Sergio Henrique Rodolpho Ramalho, Alexandra Correa Gervazoni Balbuena de Lima, Fabiola Maria Ferreira da Silva, Fausto Stauffer Junqueira de Souza, Lawrence Patrick Cahalin, Graziella França Bernardelli Cipriano, Gerson Cipriano

**Affiliations:** 1 Brazil Programa de Pós-graduação em Ciências e Tecnologias em Saúde - Universidade de Brasília, Brasília, DF - Brazil; 2 Brazil Programa de Pós-graduação em Ciências da Reabilitação - Universidade de Brasília, Brasília, DF - Brazil; 3 USA Universidade de Miami - Miller School of Medicine, Miami - USA

**Keywords:** Respiratory Insufficiency, Respiratory Muscles, Ventricular Function, Exercise Tolerance, Risk Assessment

## Abstract

**Background:**

Spirometry is underused in heart failure (HF) and the extent to which each defect associates with exercise capacity and prognosis is unclear.

**Objective:**

To determine the distinct relationship of continuous %predicted FVC (ppFVC) and FEV_1_/FVC with: 1) maximal inspiratory pressure (MIP), left ventricular ejection fraction (LVEF), exercise performance; and 2) prognosis for the composite of cardiovascular death, heart transplantation or left ventricular assist device implant.

**Methods:**

A cohort of 111 HF participants (AHA stages C/D) without diagnosed pneumopathy, spirometry, manovacuometry and maximum cardiopulmonary test. The association magnitudes were verified by linear and Cox (HR; 95% CI) regressions, age/sex adjusted. A p<0.05 was considered significant.

**Results:**

Age was 57±12 years, 60% men, 64% in NYHA III. Every 10%-point increase in FEV_1_/FVC [β 7% (95% CI: 3–10)] and ppFVC [4% (2-6)] associated with ventilatory reserve (VRes), however only ppFVC associated with MIP [3.8 cmH_2_O (0.3-7.3)], LVEF [2.1% (0.5-3.8)] and VO_2_peak [0.5 mL/kg/min (0.1–1.0)], accounting for age/sex. In 2.2 years (mean), 22 events occurred, and neither FEV_1_/FVC (HR 1.44; 95% CI: 0.97–2.13) nor ppFVC (HR 1.13; 0.89–1.43) was significantly associated with the outcome. Only in the LVEF ≤50% subgroup (n=87, 20 events), FEV_1_/FVC (HR 1.50; 1.01–2.23), but not ppFVC, was associated with greater risk.

**Conclusions:**

In chronic HF, reduced ppFVC associated with lower MIP, LVEF, VRes and VO_2_peak, but no distinct poorer prognosis over 2.2 years of follow-up. Distinctively, FEV_1_/FVC was associated only with VRes, and, in participants with LVEF ≤50%, FEV_1_/FVC reduction proportionally worsened prognosis. Therefore, FEV_1_/FVC and ppFVC add supplementary information regarding HF phenotyping.

## Introduction

Heart failure (HF) and poor lung function frequently coexist, emerging from several mechanisms: septal thickening and parenchymal congestion; impaired pulmonary vascular function and microvascular hypoperfusion; airway dysregulation and remodeling; inspiratory and peripheral skeletal muscle weakness; imbalanced chemo-, ergo- and metaboreflex for ventilatory control; heart enlargement; and decreased bronchial conductance.^1-3^ However, spirometry is largely underused in HF. Even in co-prevalent HF and in chronic obstructive pulmonary disease (COPD), 80% of the individuals performed echocardiography, but <50% undergo spirometry.^4-6^

Subclinical ventilatory alterations are present in early HF stages, contributing to dyspnea and exercise intolerance.^3^ Airway obstruction can be found mostly in non-compensated states and restrictive defects are described, particularly in chronic and stable subjects.^7,8^ It must be acknowledged that baseline spirometric defects improve cardiopulmonary exercise test (CPX) interpretation for differential diagnosis of effort limitation,^8,9^ and identify mortality risk in HF with preserved ejection fraction (HFpEF) or HF with reduced ejection fraction (HFrEF).^10,11^

However, the association of spirometry parameters with exercise limitation and prognosis in HF is still controversial,^8^ given its use in variable HF severity status and phenotypes, the possible differential contribution of each obstructive and restrictive defects, and the poorly explored potential of non-linear relationships between dynamic lung and heart dysfunctions.^12^ We hypothesized that in chronic stable HF, impaired forced vital capacity (FVC) and forced expired volume in 1 second (FEV_1_)/FVC ratio is differently associated with other functional parameters at rest and in exercise, and consequently with poorer prognosis. Therefore, we aimed to (1) define the extent to which FEV_1_/FVC and FVC are associated with left ventricular ejection fraction, respiratory strength and exercise responses; and (2) determine their associations with major incidental cardiovascular events (cardiovascular death, heart transplant and left ventricular assist device-LVAD).

## Methods

### Study Population and Clinical Characteristics

This cohort enrolled 158 consecutive HF subjects referred to the Laboratory of Physiology (Universidade de Brasília, Brasília, Brazil) for CPX from June 2015 to July 2016, followed up to at least over 24 pre-planned months. Subjects with HF, regardless of etiology or LVEF, were enrolled. They were required to be clinically stable in the previous three months (no decompensation or hospitalizations), free from diagnosed pulmonary disease (COPD, emphysema or use of bronchodilators), without medical conditions which precluded a maximal cycle-ergometer CPX. Participants had echocardiography (HD 11XE, Phillips, Amsterdam, Netherlands) done within one month from enrollment. For this analysis, we included 111 HF subjects, as spirometry data were unavailable for 43 participants, and 4 of them were unable to perform the spirometry maneuvers adequately, therefore without interpretable quality.^13^

On the first day, subjects underwent clinical evaluation, followed by respiratory strength assessment, and spirometry after a 30-minute rest. CPX was performed on the following day. Echocardiography was performed according to standard recommendations;^14^ pulmonary artery systolic pressure (PASP) was estimated from Doppler-echocardiography tricuspid regurgitation jet peak velocity, when available. Hypertension and diabetes were defined based on self-report, use of medication, or measurements at the medical appointment (blood pressure above 140/90 mmHg and fasting glucose ≥126 or random glucose ≥200 mg/dL, respectively). Dyslipidemia was defined as LDL ≥160 mg/dL or use of lipid-lowering agents. Smoking status was self-reported. The referring cardiologist informed the primary HF etiology and pharmacological prescription.

All participants signed a written informed consent and institutional review board approval was obtained from the Ethics Review Board of Universidade de Brasilia (CAAE 50414115.4.0000.0030).

### Assessment of Pulmonary Function and Respiratory Strength

Spirometry was performed according to recommendations.^13^ FEV_1_ was obtained from the volume of exhaled gas on the first second of expiration. FVC was obtained from the volume of gas vigorously exhaled after maximal inspiratory effort (Microlab, Carefusion, Yorba Linda, USA). The best of 5 forced expirations was used. Predicted reference values were derived from Brazilian equations.^15^ The continuous FEV_1_/FVC and percent predicted FVC (ppFVC) were considered the main primary exposures. As a sensitivity approach, we also analyzed terciles of each metric, and dichotomic obstructive and non-obstructive patterns (FEV_1_/FVC ≤70 and >70 respectively), and restrictive and non-restrictive patterns (ppFVC <80% and ≥80%, respectively).

Maximal inspiratory pressure (MIP) and maximal expiratory pressure (MEP) were measured according to standard recommendations,^16^ and were obtained with a digital transducer (MVD300®, Globalmed. Porto Alegre. Brazil). Subjects were sitting. and used a nose clip and a mouthpiece. MIP was determined at the maximum inspiration effort from near the residual volume, against an occluded airway with a minor air leak (2 mm). MEP was determined at the maximum expiration effort from near the vital capacity, against an occluded airway. Three to 5 reproducible (≤10% of variation between values) maneuvers were performed, sustained for at least 1 second each. They were separated by 1-minute rest and the highest value was used for analysis.^16^ Low MIP was considered when MIP was ≤ 80 cmH_2_O in men and ≤60 cmH_2_O in women.^17^

### Cardiopulmonary Exercise Test

Subjects underwent a maximum symptom-limited CPX,^18^ using cycle-ergometer ramp protocol (Corival, Lode, Netherlands) and ventilatory expired gas analysis cart (Quark CPET, Cosmed, Italy). Volume and gas calibration was performed before each test. Minute ventilation (VE), oxygen uptake (VO_2_), and carbon dioxide output (VCO_2_) were acquired breath-by-breath and averaged over 10-second intervals. The ventilatory anaerobic threshold (VAT) was determined by the V-slope method. Peak VO_2_ was expressed as the highest 10-second averaged sample obtained during the final plateau, if the patient reached it, or the highest 20-seconds average sample from the final minute of a symptom-limited test, if not. The VE/VCO_2_ slope was calculated from a linear regression equation, from the start of the test to the exercise peak. Given the fatigue reported in preliminary MVV tests (not shown) underestimating subsequent forced maneuvers or the inability to perform a sustained and reproductible measurement, particularly in patients at a more advanced stage of the disease, we were unable to use the gold-standard measured MVV uniformly to ensure comparability. Therefore, the ventilatory reserve was estimated from FEV_1_ (calculated as 100-[VE/(FEV_1_x40)100]).^18^ Circulatory power was calculated from the VO_2_ and systolic blood pressure product at peak, and ventilatory power from the quotient of peak systolic blood pressure and VE/VCO_2_ slope.^19^

### Incidental Events

The incidental endpoint was composite and included cardiovascular mortality, heart transplantation or LVAD implantation after enrollment in the study. Surveillance occurred every three months by making telephone calls, reviewing medical charts or by confirmation from local death certificate services.

### Statistical Approach

Characteristics were described using mean and standard deviation for continuous variables, and absolute numbers and percentages for categorical variables. Kolmogorov-Smirnov test was applied and continuous variables showed normal distribution. For the cross-sectional analysis, linear regression assessed the associations between FEV_1_/FVC and ppFVC exposures, and cardiac structure, respiratory strength and CPX variables as outcomes, in unadjusted and age- and sex-adjusted models, shown as ß-coefficient and 95% confidence interval (95% CI), per 10 percentage points increase in each spirometry variable. As the continuous variables were normally distributed and observations within each model were independent from each other (Pearson’s bivariate correlation coefficients <0.35 between each exposure and outcome), linear regression assumptions were verified. To address potential non-linear cardiopulmonary associations, we also tested restricted cubic spline models using 3 to 7 knots, unadjusted and age- and sex-adjusted.

For sensitivity analysis, we determined the following categories: a) sex-specific terciles of FEV_1_/FVC and ppFVC, with the first tercile representing the worst and the third tercile, the best lung function; and b) dichotomic obstructive (FEV_1_/FVC ≤70) and non-obstructive (FEV_1_/FVC>70) spirometry patterns or restrictive (ppFVC <80%) and non-restrictive (ppFVC ≥80%) patterns. Linear and logistic regressions and chi-square tests for trend were used to assess associations. To address potential asymmetries between included and excluded subjects, these groups were compared using the chi-square test for categorical variables, and the independent samples t-test for continuous variables.

For the prospective analysis, Cox regression was used to determine the magnitude of association of 10-percentage points decrease in spirometry variable with incidental composite endpoint, shown as hazard ratio (HR) and 95% CI. Non-linear associations were investigated using restricted cubic spline regression with the number of knots selected to minimize the AIC model (3 to 7 knots tested). The proportional hazards assumption was tested for all models using Schoenfeld residuals, and no violations were detected. As a sensitivity approach, four Cox regression sub-analyses were performed for each spirometric exposure, restricting them exclusively to subjects with: LVEF ≤50%; LVEF >50%; low MIP; and normal MIP.

A two-sided p-value <0.05 was considered significant for all analyses. Statistical analysis was performed using Stata software version 14.2 (Stata Corp LP, College Station, Texas, USA).

## Results

Among the 111 HF subjects, ischemic etiology was predominant, AHA stages C or D, treated according to guidelines, including 24 subjects with LVEF >50% (Table 1). Approximately half of the subjects had restrictive (ppFVC <80%) pattern, one quarter had obstructive (FEV_1_/FVC ≤70) pattern and 14 subjects (13%) had combined dysfunctions, while 40 of them (36%) had normal spirometry. From the 26 (23%) patients with body mass index (BMI) greater than 30 kg/m^2^, 15 of them (65%) showed a ppFVC <80%. Among 57 patients with ppFVC <80% (51%), 15 had BMI>30 kg/m^2^. Low MIP was a frequent finding. The average peak VO_2_ was low, assuring a maximal effort criterion. General or leg muscle fatigue were the overall limiting symptoms. No wheezing or cyanosis was observed. Five patients had ventilatory reserve lower than 20%, including 4 with 10% to 15%; they had baseline restrictive (3) or combined (2) spirometric defects and LVEF <34%. Among them, RQ range was 1.09 and 1.22. Subjects not included due to missing or poor-quality spirometry had similar characteristics as the included subjects, except for younger mean age (51.6±14.2 years). (Supplemental Table S1).

**Table 1:** – Characteristics of heart failure population at baseline (n=111). Values expressed as mean±SD or n (%)

Subjects, n	111
**Demographics and clinical characteristics**	
Age, years	57.4 ± 11.8
Male, n (%)	67 (60%)
**Etiology, n (%)**	
Chagas	32 (29%)
Ischemic	43 (39%)
Idiopathic	23 (21%)
Other	13 (12%)
BMI, kg/m^2^	26.6 ± 4.8
BMI >30 kg/m^2^; n (%)	26 (23%)
**Medical history**	
Hypertension, n (%)	63 (57%)
Diabetes, n (%)	20 (18%)
Current smokers, n (%)	29 (26%)
Dyslipidemia, n (%)	44 (40%)
NYHA, n (%)	
I	15 (13%)
II	25 (22%)
III	71 (64%)
**Medications and devices**	
Beta-blockers, n (%)	100 (90%)
ACEi/ARB, n (%)	94 (84%)
Spironolactone, n (%)	73 (66%)
Digoxin, n (%)	22 (20%)
Statin, n (%)	70 (63%)
Furosemide, n (%)	67 (60%)
Pacemaker/ICD, n (%)	25 (22%)
**Pulmonary function**	
FEV_1_, L	2.3 ± 0.7
FVC, L	3.0 ± 0.9
Percent predicted FVC, %	80 ± 17
Percent predicted FVC <80%	57 (51%)
FEV1/FVC	75 ± 9
FEV_1_/FVC ≤70	28 (25%)
MEP, cmH_2_O	84.7 ± 40.1
MIP, cmH_2_O	75.4 ± 35.4
Low MIP, n (%)	51 (49%)
**Echocardiographic characteristics**	
LVEF, %	38.4 ± 15.0
LVEF >50%, n (%)	24 (23%)
LA volume index, mL/m^2^	44.7 ± 16.7
Estimated PASP, mmHg	38.9 ± 12.0
**Cardiopulmonary test**	
Peak power, W	80.3 ± 30.6
Peak heart rate, bpm	118 ± 26
Peak systolic pressure, mmHg	151 ± 25
Absolute peak VO_2_, mL/min	966 ±401
Relative peak VO_2_, mL/kg/min	13.4 ± 4.6
RER	1.23 ± 0.18
Absolute VO_2_ at VAT, mL/min	618 ± 281
Relative VO_2_ at VAT, mL/kg/min	8.6 ± 3.5
O_2_ pulse, mL/beat	8.4 ± 3.1
OUES	1145 ± 465
VE max, (L/min)	45.0 ± 16.6
Ventilatory reserve, %	48 ± 19
VE/VCO2 slope	37.3 ± 8.1
Circulatory power, mmHg.mL/kg/min	2165 ± 1024
Ventilatory power, mmHg	4.3 ± 1.4

*BMI: body mass index; FEV_1_: forced expired volume in 1 second; FVC: forced vital capacity; NYHA: New York Heart Association functional class; ACEi: angiotensin converting enzyme inhibitors; ARB: angiotensin II receptor blockers; ICD: implantable cardioverter defibrillator; MEP: maximal expiratory pressure; MIP: maximal inspiratory pressure; LVEF: left ventricular ejection fraction; LA: left atrium; PASP: pulmonary artery systolic pressure; VO_2_: oxygen consumption; OUES: oxygen uptake efficiency slope; VE: minute ventilation; VE/VCO_2_ slope: VE/carbon dioxide production; RER: respiratory exchange ratio; VAT: ventilatory anaerobic threshold.*

### Relationship of FEV1/FVC with Functional Variables and Prognosis

Modeled continuously, FEV_1_/FVC was proportionally associated with FEV_1_ and with the ventilatory reserve from CPX, such that every 10-percentage points increase in FEV_1_/FVC, was associated with 200 mL (95% CI 100–310 mL, p<0.001) FEV_1_ increase, and with 7% points (95% CI 3–10%; p<0.001) increase in estimated ventilatory reserve, after adjusting for age and gender (Table 2). Although low MIP was a common finding in subjects with an obstructive pattern (n=15, 54%), the frequency was similar compared to the non-obstructive pattern (n=36, 47%; p=0.54), and MIP was not associated with continuous FEV_1_/FVC (p=0.90). Additionally, a non-linear association was observed between FEV_1_/FVC and FEV_1_, such that this relationship is more robust if FEV_1_/FVC is below 75% (Figure 1A). No other cardiopulmonary structure or function metric was associated with FEV_1_/FVC. These findings were also consistent across FEV_1_/FVC terciles (Supplemental Table S2).

**Table 2:** – Continuous relationship of spirometric patterns (per 10-percentage points increase in each FEV1/FVC and ppFVC) with cardiopulmonary function in heart failure subjects at baseline

Cardiac and pulmonary function		FEV_1_/FVC		% Predicted FVC	

		Coefficient (95% CI)	p	Coefficient (95% CI)	p
FEV^1^, L	Model 1	0.24 (0.10; 0.38)	0.001	0.25 (0.18; 0.31)	<0.001
	Model 2	0.20 (0.10; 0.31)	<0.001	0.23 (0.19; 0.27)	<0.001
MEP cmH_2_O	Model 1	2.7 (-6.6; 11.9)	0.57	-0.9 (-5.8; 4.1)	0.73
	Model 2	0.6 (-7.5;8.8	0.88	-1.5 (-5.8; 2.9)	0.50
MIP, cmH_2_O	Model 1	0.9 (-0.6; 8.2)	0.81	4.1 (0.2; 8.1)	0.04
	Model 2	0.4 (-6.1; 6.9)	0.90	3.8 (0.3; 7.3)	0.031
LV ejection fraction, %	Model 1	1.9 (-1.0; 4.9)	0.20	2.2 (0.6; 3.9)	0.007
	Model 2	1.6 (-1.4; 4.7)	0.28	2.1 (0.5; 3.8)	0.013
Peak power, W	Model 1	1.5 (-4.6; 7.6)	0.63	4.2 (0.9; 7.5)	0.012
	Model 2	0.3 (-4.3; 4.9)	0.89	3.5 (1.1; 6.0)	0.005
Absolute peak VO_2_, mL/min	Model 1	30 (-50; 111)	0.45	35 (-9; 78)	0.11
	Model 2	18 (-49; 86)	0.59	27 (-9; 63)	0.14
Relative peak VO_2_, mL/kg/min	Model 1	-0.3 (-1.3; 0.6)	0.47	0.6 (0.1; 1.1)	0.02
	Model 2	-0.4 (-1.3; 0.4)	0.30	0.5 (0.1; 1.0)	0.028
Respiratory exchange ratio	Model 1	-0.01 (-0.05; 0.02)	0.48	0.02 (-0.003; 0.04)	0.09
	Model 2	-0.01 (-0.05; 0.02)	0.40	0.01 (-0.003; 0.03)	0.10
Absolute VO_2_ at VAT, mL/min	Model 1	-8 (-66; 49)	0.77	12 (-20; 44)	0.45
	Model 2	-12 (-67; 44)	0.68	13 (-18; 44)	0.42
Relative VO_2_ at VAT, mL/kg/min	Model 1	-0.7 (-1.4; -0.004)	0.05	0.2 (-0.2; 0.6)	0.31
	Model 2	-0.7 (-1.4; 0.007)	0.05	0.2 (-0.2; 0.6)	0.25
O_2_ pulse, mL/beat	Model 1	0.4 (-0.2; 1.0)	0.21	0.1 (-0.2; 0.5)	0.45
	Model 2	0.4 (-0.2; 0.9)	0.18	0.1 (0.2; 0.4)	0.47
OUES	Model 1	62 (-31; 155)	0.19	19 (-32; 70)	0.47
	Model 2	48 (-34; 130)	0.25	9 (-36; 54)	0.70
VE max, L/min	Model 1	-0.4 (-3.7; 2.9)	0.82	1.6 (-0.2; 3.4)	0.08
	Model 2	-0.6 (-3.3; 2.1)	0.68	1.5 (0.02; 2.9)	0.05
Ventilatory reserve, %	Model 1	7.4 (3.9; 10.9)	<0.001	4.6 (2.8; 6.5)	<0.001
	Model 2	6.8 (3.3; 10.3)	<0.001	4.3 (2.5; 6.2)	<0.001
VE/VCO_2_ slope	Model 1	-0.2 (-2.0; 1.7)	0.87	-0.6 (-1.6; 0.3)	0.19
	Model 2	0.2 (-1.6; 2.0)	0.85	-0.5 (-1.5; 0.5)	0.35
Circulatory power, mmHg.mL/kg/min	Model 1	-20 (-202; 163)	0.83	85 (-14; 183)	0.09
	Model 2	-43 ( -212; 125)	0.61	72 (-19; 163)	0.12
Ventilatory power, mmHg	Model 1	0.08 (-0.20; 0.35)	0.59	0.12 (-0.03; 0.27)	0.11
	Model 2	0.04 (-0.22; 0.31)	0.76	0.10 (-0.04; 0.24)	0.17

*FEV_1_: forced expired volume in 1 second; FVC: forced vital capacity; MEP: maximum expiratory pressure; MIP: maximum inspiratory pressure; LV: left ventricular; VO_2_: oxygen consumption; VAT: ventilatory anaerobic threshold; OUES: oxygen uptake efficiency slope; VE max: maximum minute ventilation; VCO_2_: carbon dioxide production. Model 1: unadjusted; Model 2: age, gender. Note: p-values refer to the respective linear regression analysis.*

**Figure 1:** – Continuous association of FEV_1_/FVC (blue) and percent predicted FVC (light red) with FEV_1_, LVEF, MIP and VO_2_peak at baseline 5 using restricted cubic splines. Models were constructed using restricted cubic splines with 3 knots. *p <0.05 in models further adjusted for age and sex.

At a mean follow-up of 2.2±0.7 years, 15 subjects had cardiovascular death outcome, 3 had heart transplant and 4 had LVAD implant. Lower FEV_1_/FVC tended to increase the risk for the composite endpoint, however linearly not significant when accounting for age and sex (Table 3). Conversely, a non-linear association between FEV_1_/FVC and the composite endpoint was observed, such that the risk decreases in association with FEV_1_/FVC above 75. (Figure 2).

**Table 3:** – Association of spirometry variables at baseline with incidental composite outcome (cardiovascular mortality, heart transplant or left ventricular assist device implant; 22 events) among heart failure subjects (n=111), with mean follow-up of 2.2±0.7 years

	n	Events	Unadjusted HR (95% CI)*	p	Age/sex adjusted HR (95% CI)*	p
**FEV**_**1**_**/FVC**						
Obstructive	28	9	2.45 (1.05-5.77)	p=0.039	2.28 (0.95-5.44)	p=0.064
Non-obstructive	83	13				
Continuous	111	22	1.48 (1.00-2.18)	p=0.050	1.44 (0.97-2.13)	p=0.069
**ppFVC**						
Restrictive	57	14	1.83 (0.77-4.37)	p=0.172	1.86 (0.78-4.44)	p=0.163
Non-restrictive	54	8				
Continuous	111	22	1.16 (0.92-1.46)	p=0.207	1.13 (0.89-1.43)	p=0.306

* Per 10-unit decrease. Note: p-values refer to the respective Cox regression analysis.

**Figure 2:** – Continuous associations of FEV1/FVC (blue) and percent predicted FVC (red) at baseline with the composite outcome (cardiovascular death, heart transplant, and left ventricular assist device implant), in a mean follow-up of 2.2±0.7 years (22 events). Models were constructed for the primary exposure variables (FEV_1_/FVC and percent predicted FVC) using restricted cubic splines with 3 knots. Linear corresponds to Cox regression analysis. *p <0.05 in models further adjusted for age and sex.

Two sensitivity analyses were performed. First, excluding subjects with LVEF>50% (n=24) from the 87 remaining subjects, 20 events occurred. In this scenario, every 10-percentage points decrease in FEV_1_/FVC was associated with a 50% increase in the likelihood of the incident composite outcome per year of observation, accounting for age and sex (p=0.04) (Supplemental Figure S1). Among those with LVEF>50%, only two events occurred. Second, amongst subjects with a low MIP (n=51, 13 events), low FEV_1_/FVC was associated with heightened risk for the primary outcome (HR 1.72; 1.14-2.61; p=0.009), while in the subgroup with normal MIP (n=57, six events), it was not associated with the outcome (HR 0.98; 0.36–2.69) (Supplemental Figure S1)

### Relationship of Percent predicted FVC with functional variables and prognosis

Accounting for age and sex, every 10-percentage points increase in adjusted ppFVC was proportionally associated with a linear increase in FEV_1_, by 230 mL (95% CI 190–270 mL, p<0.001). (Table 2). MIP also increased by 3.8 cmH2O (95% CI 0.3–7.3, p=0.03), but non-linear analysis showed that such association was more robust for ppFVC <80% (Table 2 and Figure 1G). Low MIP was more frequent in HF subjects with restrictive pattern (n=34, 65%) when compared to those without restrictive pattern (n=17, 32%; p<0.001). LVEF increased by approximately 2% points for every 10-percentage points increase in ppFVC, which, also, was more prominent for ppFVC<80% (Figure 1F). Regarding CPX, the greater ppFVC, the greater peak power, relative peak VO_2_ and ventilatory power in adjusted models. No other cardiopulmonary structure or function metric was associated with ppFVC, either continuously (Table 2) or across terciles (Supplemental Table S3).

Lower ppFVC was not able to distinguish HF subjects under higher risk for the composite endpoint on the primary (Table 3 and Figure 2) or on the sensitivity analysis (Supplemental Figure S2).

## Discussion

In a real-world cohort with 111 chronic HF subjects in classes C or D, within a broad ejection fraction range, we investigated how normal-to-severe spirometric dysfunction spectrum related to resting and exercise functional metrics and to major incidental cardiovascular events. Across airway obstruction and vital capacity impairment ranges, accounting for age and sex, both low FEV_1_/FVC and ppFVC were associated with reduced exercise ventilatory reserve, but only low ppFVC was associated with low ejection fraction, inspiratory weakness, and reduced exercise capacity; and more prominent when ppFVC was lower than 80%. Although such lung dysfunctions were common, the risk for the composite endpoint of cardiovascular death, heart transplant or LVAD implant was non-linearly associated with FEV_1_/FVC only, not with ppFVC, suggesting a better prognosis in the non-obstructive pattern (FEV_1_/FVC >75%). Additionally, among the low LVEF and low MIP subgroups, only reduced FEV_1_/FVC distinguished greater risk. Therefore, FEV_1_/FVC and ppFVC differently phenotype clinical aspects of HF patients (Figure 3).

**Figure 3:** - Visual abstract of the main findings: FEV_1_/FVC and ppFVC differently characterize HF patients. HF: heart failure; LVEF: left ventricular ejection fraction; FEV1: forced expiratory volume in 1 second; ppFVC: percent predicted of forced vital capacity; MIP: maximal inspiratory pressure; CV: cardiovascular; LVAD: left ventricular assist device; HR: hazard ratio. HF: Although ppFVC was associated with other functional variables than the ventilatory reserve, only FEV_1_/FVC was associated with a relatively short-term prognosis, particularly for low ejection fraction, suggesting that each marker may add different information regarding HF phenotyping.

### Resting Lung Function and Exercise Performance Relationship

Direct HF effects on airways, such as vascular congestion, parenchyma and alveolar edema and interstitial fibrosis, are related to acute/subacute airway diameter constriction and to subacute/chronic lung volume reductions.^1-3^ As a result, FEV_1_ and FVC decrease independently or concurrently, suggesting that even subclinical dysfunction could contribute differently to exercise intolerance. Accordingly, FEV_1_ was more robustly associated with aerobic capacity (peakVO_2_) than LVEF.^8,20^ Therefore, the maximum voluntary ventilation (derived from FEV_1_), is expected to be impaired in HF, proportionally to underlying severity of lung dysfunction. However, exercise intolerance in HF subjects is multifactorial and reduced ventilatory reserve at peak only partially expresses lung contribution.^7^ Indeed, we observed that ventilatory reserve increased 7% and 4% points for every 10% points increase in FEV_1_/FVC and ppFVC, respectively. However, ventilatory reserve was as low as 38% in FEV_1_/FVC and 39% in the lowest terciles of ppFVC on average, which is greater than the 20% expected to unequivocally assume a ventilatory constraint in peak exercise, supporting the cardiocirculatory limitation as to the primary – but not unique – effort limitation origin in our subjects.

Parameters such as OUES (Oxygen Uptake Efficiency Slope) and VE/VCO_2_ slope are less dependent on peak effort and are more sensitive to distinguishing ventilatory from cardiocirculatory constraints.^20,21^ The average values of low OUES, high VE/VCO_2_ slope and low O_2_ pulse in our study actually suggest a cardiocirculatory limitation predominantly, but the lack of association between resting FEV_1_/FVC and ppFVC with other exercise ventilatory variables, including ventilatory efficiency, was contrary to our initial hypothesis. Therefore, the ability of resting spirometry to precisely measure ventilatory contribution to exercise impairment, other than ventilatory reserve, may be limited, overwhelmed by the cardiocirculatory component in more advanced HF stages, as in our cohort, with patients at stages C/D, predominantly in NYHA III (64%) and mean peakVO2 of 13 mL/kg/min.^22^

Regarding other functional variables, only ppFVC, not FEV_1_/FVC, was additionally associated with resting MIP, but not MEP, with LVEF and with peak power and peak relative VO_2_, accounting for age and sex.

Given the severe yet stable characteristics of HF subjects in this study, the discrepant correlations for each exposure could possibly result from the primary influence of HF in reducing total lung capacity, disproportionally decreasing FVC relative to FEV_1_, therefore attenuating the FEV_1_/FVC effect to predict exercise responses.^1^ Accordingly, a restrictive pattern is usual in the HF syndrome,^1,7,8^ particularly in HFrEF.^23^ Additionally, the direct relationship of ppFVC with LVEF supports the hypothesis that a potential enlarged and dysfunctional heart relates to the aforementioned reduced lung volume due to space-occupying and congestive vascular and parenchymal effects. Such alterations, aggravated by inspiratory weakness, can compromise breathing mechanics in response to increasing demands, further reducing lung compliance, and all may contribute to exercise limitation, represented by the associated low-peak VO_2_.^1,7,8^

Interestingly, only ppFVC was associated with the MIP. In HF, reduced vital capacity is associated with low MIP and diaphragm dysfunction,^24,25^ which plays a significant role in exercise limitation in HFrEF^26,27^ and HFpEF,^28^ and demonstrates independent prognostic relevance.^29^ Consistent with our findings, generalized skeletal muscle dysfunction and structural abnormalities, particularly the diaphragm, largely contributes to exercise intolerance in both HFrEF and HFrEF.^30^ Automaticity and constant work overload, even at rest, uniquely characterize the diaphragm predisposition to early dysfunction in HF syndrome more prominently than expiratory and other peripheral muscles.^31^

### Lung Function and Cardiovascular Prognosis

Lung function decline, beginning subclinically, have shown an association with incidental heart failure in unselected general populations.^32,33^ Additionally, in subjects with stable HFrEF, spirometry significantly predicted all-cause mortality.^34^ Olson et al.^34^ studied 134 HFrEF subjects with peak VO_2_ of 19 mL/kg/min and 66% NYHA classes I and II, and showed that lower FEV_1_ and FVC had lower survival rates. In contrast, in advanced pre-transplant HFrEF, Georgiopoulou et al. demonstrated that spirometry provided no prognostic information.^35^ In our cohort, positioned between the previous studies regarding severity, continuous FEV_1_/FVC and ppFVC were not able to distinguish HF subjects with increased risk for major cardiovascular events in linear models.

Probably, given the chronic HF stages and the predominant cardiocirculatory limitation from the CPX, as demonstrated, ventilatory impairment contribution provided minor additional prognostic information for the whole LVEF range HF. Attempting to address a potential dynamic relationship of such complex biological interaction, we investigated non-linear associations for possible ranges or thresholds under differential risk across the spectrum of both exposures. We found a non-linear association between FEV_1_/FVC and the composite endpoint, in which HF subjects with FEV_1_/FVC greater than 75% decreased the likelihood of having major cardiovascular events in a mean follow-up of 2.2 years. We can hypothesize that: 1) COPD, the leading cause of airway obstruction which frequently coexists with HF, could have been undetected, increasing the risk burden;^4^ or 2) from primary HF effects on respiratory system, the FEV_1_ changes may be more sensitive than FVC in shorter time periods, influenced by dynamic changes in small and mid-bronchi calipers.^23^ Reduced vital capacity, a hallmark of advanced HF,^9^ was less sensitive in distinguishing the risk of incidental events throughout this follow-up.

Potential contributions from lung function to incidental cardiovascular events also appear to differ between HFpEF and HFrEF phenotypes. Restricting the analysis to a subset of subjects with LVEF ≤50%, a decrease in FEV_1_/FVC, but not across the ppFVC spectrum, identified a higher risk for the composite endpoint, while no conclusion could be made for those with LVEF >50% with only two events. Similarly, in the inspiratory weakness subset, decreasing FEV_1_/FVC distinguished greater risk for major cardiovascular events, which was not observed among those without weakness or across the ppFVC spectrum. We could speculate that the obstructive pathophysiology pattern adversely impacted mostly those with reduced LVEF and with inspiratory weakness.

### Limitations

Several limitations should be noted. As an observational study, causality could not be addressed, and residual and unmeasured confounders may exist for the observations described. Only a subset of subjects was included, with complete spirometry, necessary for this study, and, given the younger age of excluded subjects, involuntary selection bias could be present. Spirometry measures were performed without bronchodilators, so reversible obstruction remained undetected; additionally, restrictive patterns were based only on FVC, because more precise and direct measures of volumes and capacities were unavailable, which could have limited the ability to detect true lung volume restriction, but could increase the external validity of findings. Also, an important mechanism of exercise limitation could be due to air trapping, which cannot be detected by spirometry.

Unfortunately, measured MVV was unfeasible to all participants, which could have influenced ventilatory reserve metrics, most likely underestimated. Even so, only 5 participants had <20% ventilatory reserve. However, we traded off potential unreliable results for uniform and comparable values throughout the cohort using an estimated MVV measure. Despite a reasonable correlation between FEV_1_ and MVV (r^2^=0.82),^36^ we acknowledge that MVV must be performed prior to CPET at the individual level whenever possible.

We also acknowledge that low exercise oxygen saturation may represent ventilation-perfusion mismatch or ventilatory restraint, which is not exclusively, but most frequently associated with respiratory system impairment, particularly advanced COPD, interstitial lung diseases and pulmonary hypertension,^18^ conditions excluded at study enrollment. However, an oximeter compatible to our CPX system was unavailable during data acquisition and the existing fingertip probe produced unreliable peak values. Therefore, we resorted to the unremarkable physical exam (no peak exercise wheezing or cyanosis) to assume that hypoxia was unlikely.

Lastly, the relatively short follow-up time and, consequently, event rate, may have limited the ability to detect prognostic associations with ppFVC rather than FEV_1_/FVC, given the more chronic behavior of the former.

### Implications for Clinical Practice

Spirometry and manovacuometry are extensively available pulmonary function tools, although underused in HF.^4,26^ Interpretation of ventilatory defects can be challenging in these subjects, particularly in those with HFpEF, in whom phenotype variation can potentially overlap heart and lung symptoms and fewer data are available.^9^ However, they can provide valuable information on HF impact on the respiratory system, differentiating from undiagnosed (and undertreated) lung disease, better interpreting CPX, identifying potential therapeutic targets (rehabilitation, ventilatory training) and defining prognostic risk factors, emphasizing that spirometry is an available and feasible tool, which must be performed prior to CPX and to support risk stratification in HF. Subclinical and early stages alterations in lung function may predict future cardiovascular events. Adding to that knowledge, our study suggests that also in more chronic and stable HF, the presence and type of lung dysfunction help to better interpret exercise responses and to identify subjects at higher risk.

## Conclusion

In a real-world cohort with chronic HF subjects, irrespective of ejection fraction range, continuous FEV_1_/FVC and ppFVC at baseline were directly associated with ventilatory reserve at exercise, accounting for age and sex. However, only low ppFVC was additionally associated with low ejection fraction, inspiratory weakness, and reduced exercise capacity. Within a 2.2-year mean follow-up, only FEV_1_/FVC, but not ppFVC, distinguished HF subjects at higher risk for major cardiovascular events, which were more prominent among those with reduced ejection fraction and low inspiratory pressure. Therefore, FEV_1_/FVC and ppFVC add different information regarding HF phenotyping.
